# Antibacterial Peptide NP-6 Affects *Staphylococcus aureus* by Multiple Modes of Action

**DOI:** 10.3390/ijms23147812

**Published:** 2022-07-15

**Authors:** Xiaoyan Hou, Jianlong Li, Huaqiao Tang, Qingye Li, Guanghui Shen, Shanshan Li, Anjun Chen, Zixin Peng, Yu Zhang, Chaowei Li, Zhiqing Zhang

**Affiliations:** 1 College of Food Science, Sichuan Agricultural University, Ya’an 625014, China; xiaoyanhou@sicau.edu.cn (X.H.); jlli999@foxmail.com (J.L.); qingyeli@sicau.edu.cn (Q.L.); shenghuishen@163.com (G.S.); lishanshan.812@163.com (S.L.); anjunc003@163.com (A.C.); pzx18227584098@163.com (Z.P.); zhangyu2039@163.com (Y.Z.); lichaoweiyx@163.com (C.L.); 2 College of Veterinary Medicine, Sichuan Agricultural University, Chengdu 611137, China; turtletang@163.com

**Keywords:** antibacterial peptide, *Staphylococcus aureus*, modes of action

## Abstract

Our previous study extracted and identified an antibacterial peptide that was named NP-6. Herein, we investigated the physicochemical properties of NP-6, and elucidated the mechanisms underlying its antimicrobial activity against *Staphylococcus aureus*. The results showed that the hemolysis activity of NP-6 was 2.39 ± 0.13%, lower than Nisin A (3.91 ± 0.43%) at the same concentration (512 µg/mL). Negligible cytotoxicity towards RAW264.7 cells was found when the concentration of NP-6 was lower than 512 µg/mL. In addition, it could keep most of its activity in fetal bovine serum. Moreover, transmission electron microscopy, confocal laser scanning microscopy, and flow cytometry results showed that NP-6 can destroy the integrity of the bacterial cell membrane and increase the membrane permeability. Meanwhile, NP-6 had binding activity with bacterial DNA and RNA in vitro and strongly inhibited the intracellular β-galactosidase activity of *S. aureus*. Our findings suggest that NP-6 could be a promising candidate against *S. aureus*.

## 1. Introduction

Foodborne pathogenic bacteria are potential hazards in the entire production and marketing process of food and edible agricultural products, which poses a serious threat to food safety. Notably, *Staphylococcus aureus* is one of the most harmful and widely distributed foodborne pathogens. Although traditional antibiotics prevent and control pathogenic microorganisms, the abuse of antibiotics in recent decades has led to an increasing number of drug-resistant bacteria, which seriously threatens human and animal health, and even ecological security.

Therefore, this calls for urgent identification of new agents against antibiotic-resistant bacteria. Antibacterial peptides (AMPs) are important effector molecules of the innate immune system. AMPs have received increasing attention because they are a new type of broad-spectrum antimicrobial drugs that are not prone to drug resistance. According to The Antimicrobial Peptide Database [1], approximately 3250 antimicrobial peptides have been reported since Hultmark et al. [2] discovered cecropin from the giant silk moth *Hyalophora cecropia*. AMPs have numerous advantages in addition to the massive range of AMPs sources, including their small relative molecular weight (usually 30~60 amino acids), strong cationic charge, good thermal stability, acid-base stability, and broad-spectrum ability. Studies have reported that AMPs could not only kill or inhibit the invasion of pathogens, but also have antiviral, cell healing promotion, immune regulation, and other functional properties [3]. This suggests that AMPs have broad application prospects in medicine, breeding, and food industries.

AMPs are widely considered as a new type of antimicrobial agent that are expected to replace antibiotics in the prevention and control of pathogenic microorganisms. However, the exact mechanism of antimicrobial peptides has not yet been elucidated, which limits their application. It has been reported that they can inhibit or kill microbes by disrupting cell membrane integrity, inhibiting the synthesis of protein, DNA, RNA, and (or) other intracellular targets [4,5]. Moreover, the positive charge, hydrophobicity, and composition of amino acids contribute directly to the antimicrobial potency of AMPs [6]. For most AMPs, the existence of a positive charge helps them to bind with the cell membrane of a target microbe, making them permeable or formatting pores [7]. Several models have been proposed to describe the interaction between AMPs and microbial membranes.

An accumulation of AMPs leads to the formation of a double-layered hydrophilic antibacterial peptide molecular wall, similar to a carpet. Therefore, the mechanism is referred to as the “carpet model”, and peptides such as PGLa [8] and BP100 [9] act in this mode. However, AMPs such as magainins [10], mastoparan X [11], and buforin 2 [12] work in another mode (toroid pore model). While in the “toroid pore model”, the membrane phospholipid bilayer inflects after combining with the hydrophobic terminal of AMPs, thereby inducing the formation of a pore that is composed of hydrophobic groups of AMPs and phospholipid molecules. At the same time, AMPs enter the cytoplasm and may target intracellular components. In contrast, the “barrel-stave model” is proposed as follows: AMPs with an α–helix conformation aggregate on the membrane surface and insert into the membrane in a perpendicular form to the surface of the bilayer [13]. The amphiphilicity of AMPs plays a vital role in this model [13]. Studies have shown that AMPs such as alamethicin [14] and pardaxin [15] can form a barrel channel.

However, some AMPs can also act on the intracellular targets [16,17,18]. Several studies have reported that AMPs can inhibit DNA replication and RNA synthesis, affect protein synthesis, and inhibit the formation of cell walls and the activity of intracellular enzymes, ultimately leading to cell death [19,20]. In many cases, the mechanism of membrane damage and intracellular action of AMPs usually complement each other in the exertion of antibacterial activity [21].

In our previous research, a novel antimicrobial NP-6 was successfully identified by bioinformatics and proteomics from Sichuan pepper seeds, and its minimum inhibitory concentration (MIC) against *Staphylococcus aureus* (*S. aureus*) was 512 µg/mL [22]. In the present work, more information about NP-6 was investigated and discussed. Firstly, the physicochemical and biological properties of NP-6 were predicted by online software. Moreover, the influence of NP-6 on the cell membrane of *S. aureus* was deeply discussed by means of transmission electron microscopy, flow cytometry, and laser confocal microscopy. Additionally, the interaction between NP-6 and bacterial nucleic acid was investigated.

## 2. Results and Discussion

### 2.1. Physicochemical Properties of NP-6

Prediction approaches are useful ways to discover characters of novel AMPs, predict the mode of action, or to suggest a likely three-dimensional (3D) structure when no experimental conformational data are available [23]. The 3D structure has been investigated in our previous report. In this section, we further predict the physicochemical parameters of NP-6. The predicted net charge, half-life, GRAVY (Grand average of hydropathicity) score, and amphiphilicity of NP-6 are shown in Table S1.

The possibility for NP-6 to form aggregation was predicted based on the energy states, hydrophobicity, solvation energetics, electrostatic interactions, and hydrogen bonding of peptides [24]. The results are shown in Figures S1 and S2. In addition, the time-killing curve of NP-6 against *S. aureus* was shown in Figure S3.

#### 2.1.1. Hemolysis Activity of NP-6

**Figure 1:**
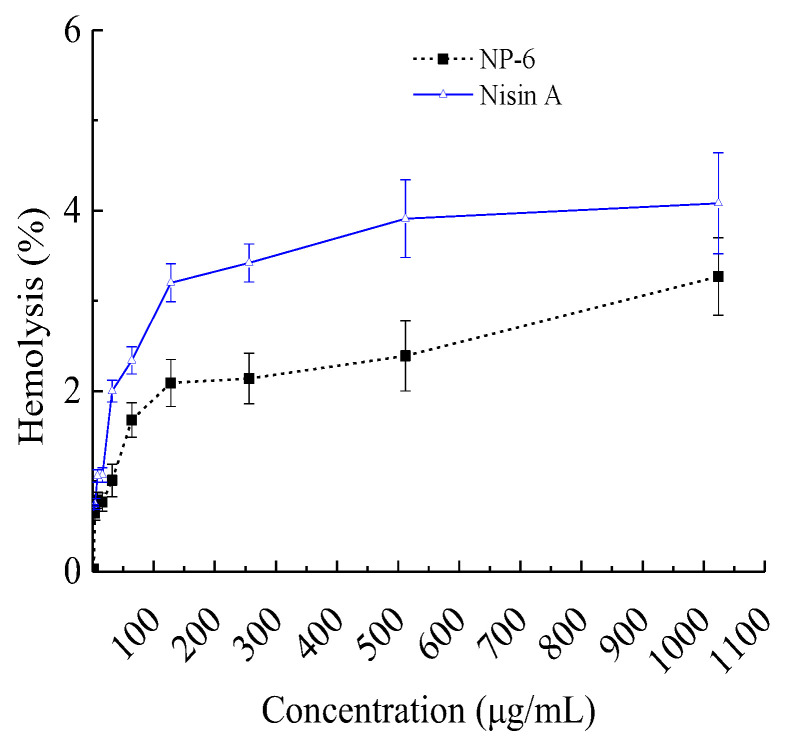
Hemolysis activity of NP-6. The values represent the means ± SD (*n* = 3).

When they are used as a preservative or antibiotic substitute, AMPs with improved antibacterial activity but decreased hemolytic activity of mammalian cells are of great importance. In view of this, we investigated the hemolytic activity of NP-6, and the results are shown in Figure 1. It suggested that the hemolysis activity of NP-6 increased with its concentration. At 512 µg/mL (1MIC), the hemolysis activity of NP-6 was 2.39 ± 0.13%, lower than Nisin A (3.91 ± 0.43%) at the same concentration. As was reported by Goudarzi et al. [25], the standard non-hepatotoxicity grade was obtained when the hemolytic percentage was lower than 5%. Higher hydrophobicity was correlated with stronger hemolytic activity and there have been many reports on how to reduce the hemolytic activity of antibacterial peptides. Zhao et al. [26] found that the Arg/Trp substitutions can enhance the affinity of the peptides to anionic lipids, but not neutral lipids, resulting in enhanced antimicrobial and reduced hemolytic activity. In addition, Sahariah et al. [27] reported that grafting of antimicrobial peptide to chitosan polymers is a strategy for abolishing the hemolytic propensity and increasing the activity of the parent peptide.

It had been reported that mammalian cell membranes have a greater concentration of cholesterol which is likely to weaken the hydrophobic interactions with AMPs [26]. Despite this, a few numbers of AMPs have been confirmed to be cytotoxic to mammalian cells, suggesting that it is important to investigate the interactions of AMPs with mammalian cells as well as with bacterial ones in order to develop them into attractive drug candidates.

#### 2.1.2. Cytotoxicity of NP-6

**Figure 2:**
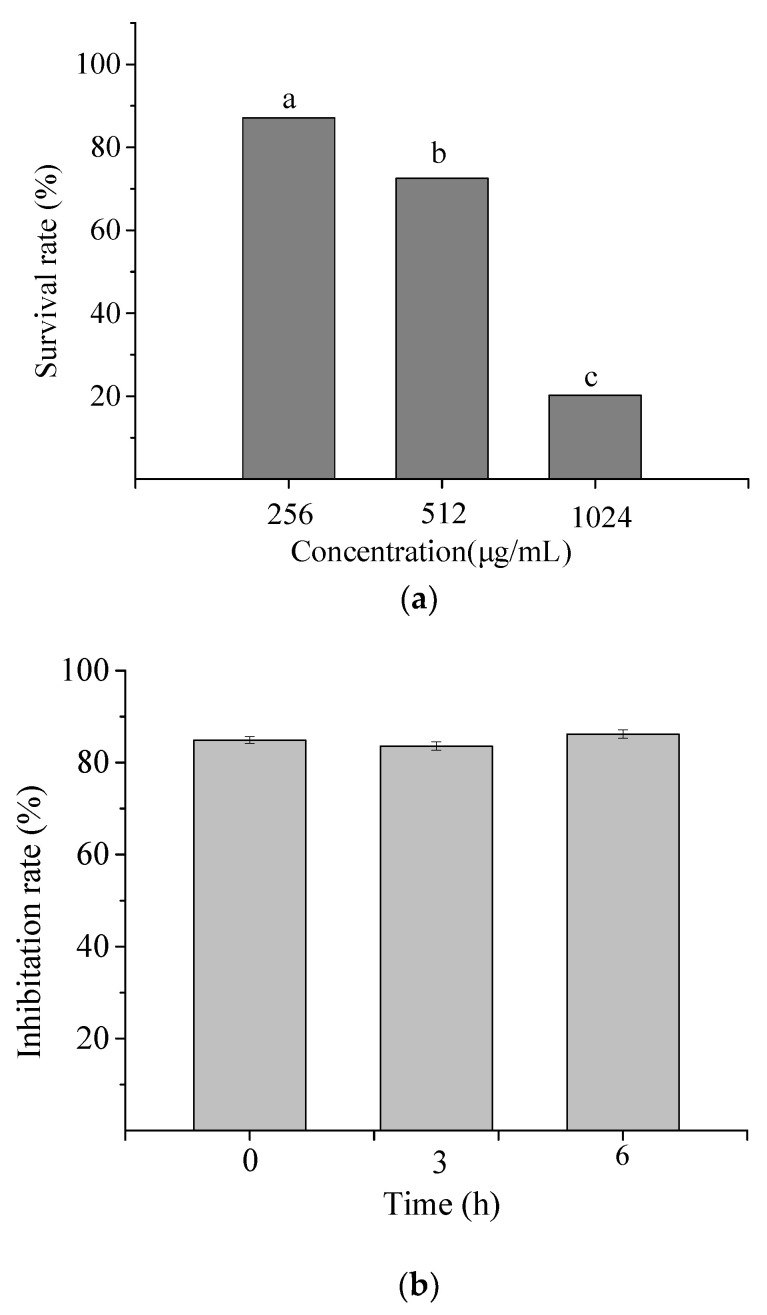
Cytotoxicity (**a**) and serum stability (**b**) of NP-6. The values represent the means ± SD (*n* = 3). Bars with different letters are significantly different (*p* < 0.05).

The toxic effect of NP-6 on RAW264.7 cells was measured and the result is shown in Figure 2a. At 1/2 MIC and 1 × MIC (512 µg/mL), the survival rate of RAW264.7 cells was 87.11% and 72.56%, respectively. While the value sharply reduced to 20.26% when the concentration increased to 2 × MIC (1024 µg/mL). The results indicated that NP-6 had negligible cytotoxicity towards RAW264.7 cells at MIC or concentrations that were lower than MIC.

#### 2.1.3. Serum Stability of NP-6

Figure 2b shows the antibacterial activity of NP-6 after incubation with serum for different times. According to the figure, the inhibition rate of NP-6 + serum samples all exceeded 80% relative to NP-6, suggesting that NP-6 could keep most of its activity in serum.

### 2.2. TEM Observations

**Figure 3:**
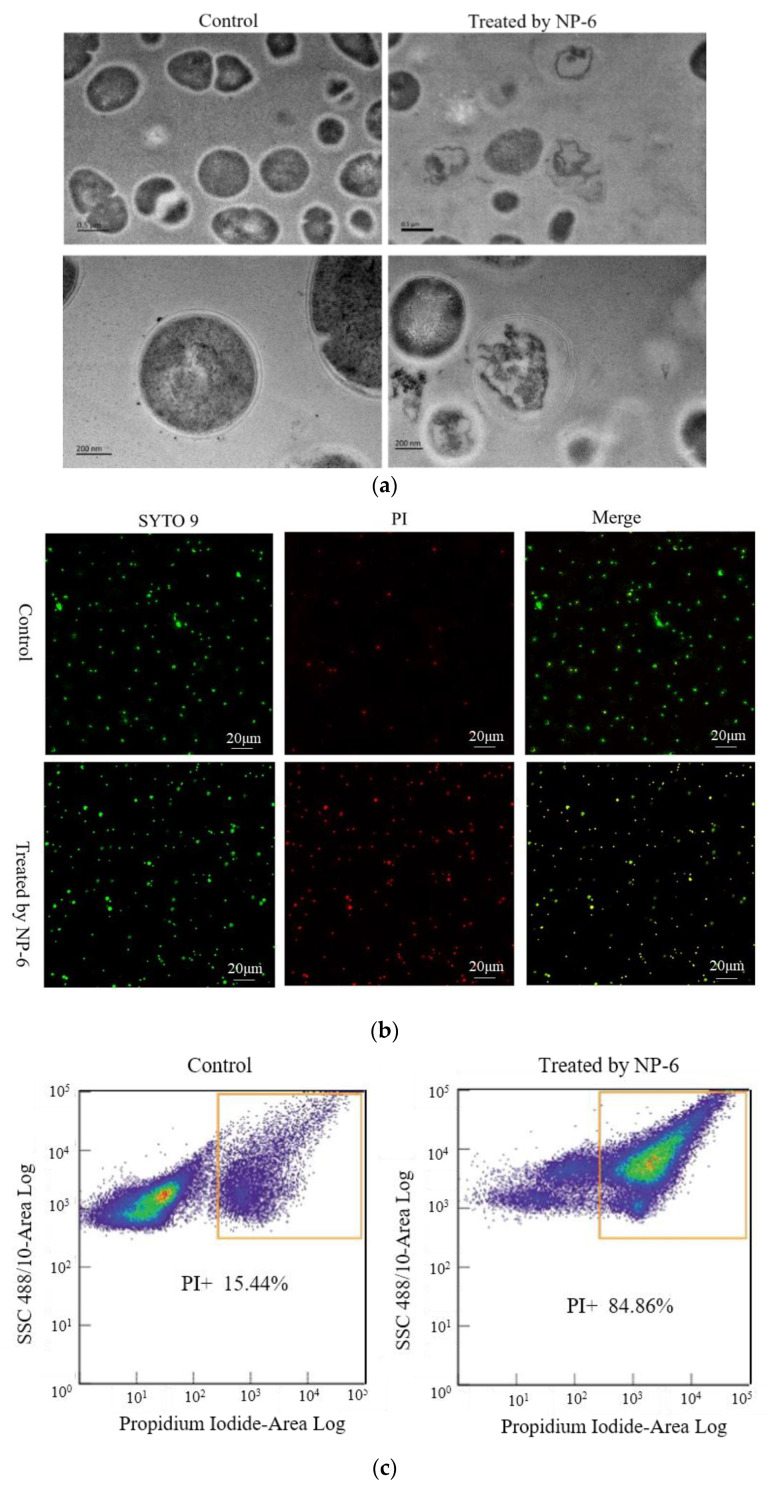
Effect of NP-6 on the cell membrane of *S. aureus*. (**a**)Transmission electron micrographs; (**b**) confocal images; (**c**) flow cytometry analysis. The control was processed without NP-6. Note: In (**b**), green dots indicate cells are either alive or dead, red dots indicate dead cells, yellow dots indicate dead cells.

The changes of internal substance and cell membrane of bacteria can be observed clearly through the transmission electron microscope. The TEM images of *S. aureus* membrane that was incubated with/without NP-6 are shown in Figure 3a. According to the figure, bacteria cells that were incubated in the absence of NP-6 have a smooth membrane surface, integrated structure, and the contents are complete. By contrast, bacteria that were exposed to NP-6 exhibit damaged cell membranes and reduced cytoplasm, suggesting that NP-6 has the ability to break the cell membrane of *S. aureus*. The result is consistent with observations that were conducted in *E. coli* in our previous work [28].

Membrane destruction is one of the most reported mechanisms of AMPs, which can be observed using a transmission electron microscopy (TEM). Xu et al. [29] conducted TEM observations of *S. aureus* and found that it was rough, denatured, and full of bubbles after treatment with two kinds of β-sheet AMPs. Wang et al. [30] found that temporin-1 Cea could cause a significant change in the cell membrane of BCAP-37. However, TEM alone is not enough, more assays should be done to confirm the observation.

### 2.3. PI Uptake Analysis

PI is a cell-impermeable dye that emits fluorescence upon binding to nucleic acids. It can penetrate the cells with a destroyed membrane and stain them [31]. 

Herein, we captured CLSM images of bacteria cells that were stained by SYTO 9 and propidine iodide (PI). *S. aureus* samples that had not been incubated with NP-6 were used as the control. As shown in Figure 3b, green dots in the left column indicate cells that are either alive or dead, red dots in the middle indicate dead cells, yellow dots on the right indicate dead cells, and green dots indicate cells that are alive. It was apparent that only a small amount of *S. aureus* was stained by PI in the control group. In contrast, most of the bacterial cells that were treated with NP-6 stained red, indicating that NP-6 can increase the permeability of *S. aureus* cell membrane.

Flow cytometry was further conducted to verify this. As shown in Figure 3c, the percentage of bacteria cells that were stained by PI was 84.86% after incubation with NP-6 for 1 h, while it was only 15.44% in the absence of NP-6. The results indicate that NP-6 increased the permeability of bacterial cell membranes, leading to increased PI-labeled cells. According to our previous work [28], NP-6 induced more PI-labeled cells of *E. coli*, indicating that NP-6 exhibits higher activity against *E. coli* than *S. aureus*. This also can be confirmed by MIC of NP-6 against the two different bacteria [22].

Damage to the cell membrane is considered to be one of the main mechanisms of AMPs. Various researchers have found that antibacterial peptides have the ability to increase membrane permeability. According to previous studies, AMPs might bind preferentially to the negatively charged phospholipids of the membrane due to the presence of positively charged residues [31]. It is likely that NP-6 interacted with the negatively charged head groups of the membrane phospholipids and subsequently rotated, allowing the hydrophobic residues (such as Ala and Leu) to interact with the membrane and increasing the membrane permeability.

### 2.4. Binding Ability of NP-6 with Bacterial DNA and RNA

**Figure 4:**
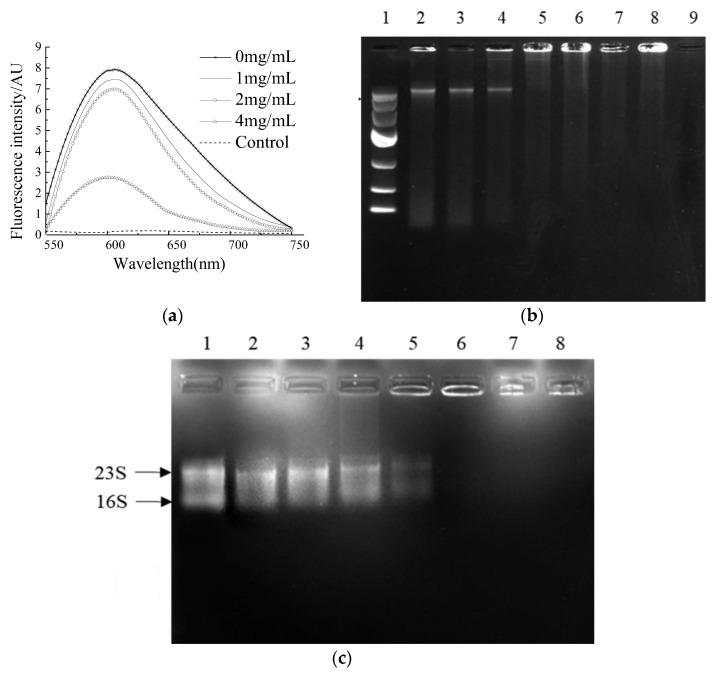
The binding ability of NP-6 with bacterial DNA/RNA. (**a**) Competitive binding of NP-6 and EB with *S. aureus* genomic DNA. Fluorescence spectra were measured from 550 to 750 nm; (**b**) Agarose gel electrophoresis of peptide and bacterial genomic DNA. Lane 1: DNA marker; lane 2: control, without peptide; lane 3: 0.1 mg/mL peptide; lane 4: 0.2 mg/mL peptide; lane 5: 0.4 mg/mL peptide; lane 6: 0.8 mg/mL peptide; lane 7: 1.0 mg/mL peptide; lane 8: 2.0 mg/mL peptide; lane 9: 4.0 mg/mL peptide; (**c**) Agarose gel electrophoresis of peptide and bacterial RNA. Lane 1: control, without peptide; lane 2: 0.1 mg/mL peptide; lane 3: 0.2 mg/mL peptide; lane 4: 0.4 mg/mL peptide; lane 5: 0.8 mg/mL peptide; lane 6: 1.0 mg/mL peptide; lane 7: 2.0 mg/mL peptide; lane 8: 4.0 mg/mL peptide.

A comparison of the binding ability of NP-6 and EB with bacterial genomic DNA was conducted in vitro. EB is a highly sensitive nucleic acid dye, which will produce fluorescence that is 20~30 times higher than its own when it binds to DNA. As was shown in Figure 4a, the fluorescence intensity of EB-DNA system decreased with the increasing concentration of NP-6, indicating that EB was replaced by NP-6. Compared to EB, NP-6 has a stronger binding ability with bacterial DNA.

DNA gel retardation was subsequently conducted to confirm the binding activity of NP-6 with bacterial DNA. The results showed that the brightness of a DNA strip in agarose gel electrophoresis reduced as the concentration of NP-6 increased (Figure 4b). DNA strips that were treated with NP-6 at 0.4, 0.8, 1.0, and 2.0 mg/mL were almost trapped in the spotting hole, suggesting that NP-6 had DNA-binding activity in vitro. 

The interaction of NP-6 and bacterial RNA was further evaluated using a gel retardation assay. According to results that are shown in Figure 4c, as the NP-6 concentration increased, the RNA band was closer to the spotting hole and its color gradually darkened. Moreover, the migration of bacterial RNA was almost completely suppressed in the spotting hole, which suggested that NP-6 can bind with bacterial RNA in vitro. 

It was previously reported that the attraction between the positively charged AMPs and the negatively charged DNA of bacteria may lead to the accumulation of peptides in bacterial cells [32]. Cardoso et al. [5] found that Buforin II could combine with DNA without causing damage to the cell membrane. Liu et al. [33] investigated the mechanism of an amide-modified peptide CecXJ-37N, and found that it could non-covalently intercalate into the nucleotides of bacterial genomic DNA. The DNA and RNA binding experiments of NP-6 further proved that DNA/RNA might be the intracellular targets in cells.

### 2.5. Effect of NP-6 on the Activities of Intracellular T-ATPase and β-Galactosidase

**Figure 5:**
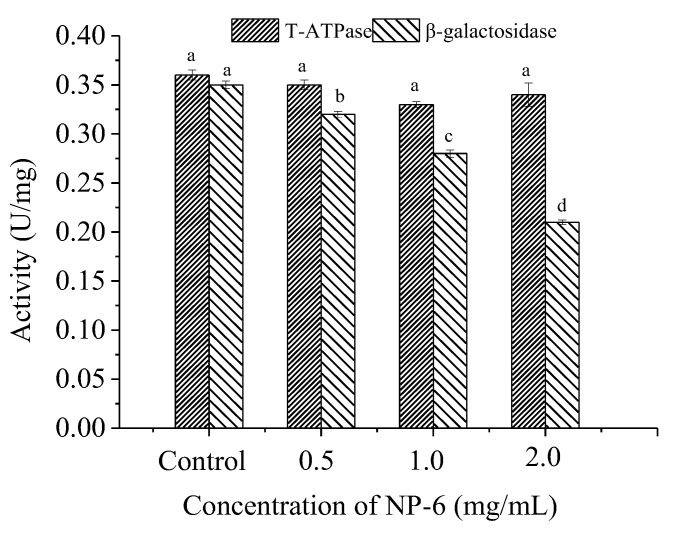
The effect of NP-6 on the activities of bacterial intracellular enzymes. The values represent the means ± SD (*n* = 3). Bars with different letters are significantly different (*p* < 0.05).

According to Figure 5, there is no significant (*p* > 0.05) change in T-ATPase (total adenosine triphosphatase in the cell) activity of bacteria, indicating that NP-6 treatment has little effect on ATPase activity of *S. aureus*. However, it was observed that the peptide strongly inhibited the intracellular β-galactosidase activity of *S. aureus* in a dose-dependent manner (*p* < 0.05). The results indicated that intracellular enzymes may also be targets of NP-6 to exert antibacterial activity. 

## 3. Material and Methods

### 3.1. Materials

NP-6 and Nisin A was chemically synthesized by GL Biochemistry Corporation (Shanghai, China), followed by further purification with a RP-HPLC system and characterization with ESI-MS (Waters, Madrid, Spain), yielding a final purity of the peptide of more than 95%. 

Nutrient broth (NB) and nutrient agar (NA) were purchased from Hangzhou Microbial Reagent Co., Ltd. (Hangzhou, China). SYTO 9 (S34854) was purchased from Molecular Probes (Invitrogen, Barcelona, Spain). Propylene iodide (PI) was purchased from Sigma (St. Louis, MO, USA). M9 medium, ethidium bromide (EB), and β-galactosidase activity assay kit were purchased from Sangon Biotech Co., Ltd. (Shanghai, China). DNA/RNA extraction kits were purchased from Tiangen Biotech Co., Ltd. (Beijing, China). T-ATPase test kit was purchased from Nanjing Jiancheng Bioengineering Institute (Nanjing, China). Dulbecco’s modified Eagle’s medium (DMEM) medium and fetal bovine serum (FBS) were all purchased from Gibco^®^ by Life Technologies (Grand Island, NY, USA).

*Staphylococcus aureus* (*S. aureus*, ATCC 25923) was kindly provided by the Microbiology Laboratory, College of Food Science, Sichuan Agricultural University, cultured in sterilized NB (peptone, 10 g/L, yeast extract, 5 g/L, NaCl, 10 g/L) at 37 °C. 

RAW 264.7 cells were purchased from the National Collection of Authenticated Cell Cultures (Shanghai, China). The cells were cultured in DMEM containing 10% FBS at 37 °C in a 5% CO_2_ incubator at constant temperature.

### 3.2. Physicochemical Properties of NP-6

The prediction of physicochemical properties and structural characteristics of NP-6 were based on its primary sequence consisting of 19AA (AGDKKIKIGINGFGRIGRL). High performance liquid chromatography and mass spectrometry of NP-6 were shown in Figure S4. The net charge, hydrophobicity, and amphipathic of NP-6 were predicted by Protparam (https://web.expasy.org/protparam/) of ExPASy (accessed on 15 January 2022) [34]. The structural fluctuations of NP-6 was evaluated by CABS-flex server (http://biocomp.chem.uw.edu.pl/CABSflex2/index) (accessed on 15 January 2022) [35]. Aggregation potentiality of NP-6 in solution and in the presence of bacterial membrane were measured by Tango (http://tango.crg.es/) (accessed on 15 January 2022) [36]. The orientation and potential interaction of NP-6 with lipid membrane were studied by Orientation of Proteins in Membrane (OPM) webserver (https://opm.phar.umich.edu/ppm_server) (accessed on 15 January 2022) [37]. 

#### 3.2.1. Hemolysis Assay

The hemolytic activity of the peptide was evaluated according to Tang et al. [38] with minor modifications. To prepare the red blood cell solution, 1.0 mL of fresh mouse red blood cells was centrifuged for 5 min at 1000× *g*, and the precipitates were resuspended in 10 mL of PBS (0.01 M, pH 7.4). A total of 50 µL of serial dilutions of peptides (2048, 1024, 512, 256, 128, 64, 32, 16, 8, and 4 µg/mL) were prepared in a 96-well plate by dissolving NP-6 and Nisin A in PBS (0.01 M, pH 7.4). Then, 50 µL of freshly prepared mouse red blood cell solution was added and incubated at 37 °C for 1 h. A total of 50 µL of PBS buffer only and 50 µL of Triton X-100 1% (*w*/*v*) were mixed with 50 µL of mouse red blood cell solution as negative and positive controls, respectively. After 1 h of incubation, all the samples were centrifuged at 1000× *g* for 5 min. The supernatant was collected, and the absorbance was at 595 nm with a UV–vis instrument at 25 °C. The percent hemolysis was calculated as the following equation:

(1) $$\%\mathrm{hemolysis}=\frac{{\mathrm{absorbance}}_{\mathrm{sample}}-{\mathrm{absorbance}}_{\mathrm{negative}}}{{\mathrm{absorbance}}_{\mathrm{positive}}-{\mathrm{absorbance}}_{\mathrm{negative}}}\times 100$$

Mouse red blood cells that were suspended in PBS and 1% Triton X-100 represented zero hemolysis and 100% hemolysis, respectively [39].

#### 3.2.2. Cytotoxicity Assay

The Cell Counting Kit-8 (CCK-8) assay was conducted to measure the cytotoxicity of the peptides towards RAW 264.7 cells according to Deng et al. [40]. The cells were incubated in Roswell Park Memorial Institute (RPMI)-1640 medium at 37 °C in a humidified atmosphere with 5% CO_2_. Then, they were added to a 96-well plate (3 × 10^3^ cells/well) to adhere for 18 h. A total of 100 µL of the samples with different concentrations (256, 512, 1024, and 2048 µg/mL) diluted by H_2_O were added and incubated for another 48 h, after which the medium was disposed. A total of 100 µL of the CCK-8 test solution was added and incubated for 2 h at 37 °C. The absorbance values of the mixture at 450 nm were determined. The survival rate of cells was calculated according to the following formula:

(2) $$\;\mathrm{Survival}\;\mathrm{rate}\;\left(\%\right)=\frac{{\mathrm{absorbance}}_{\mathrm{sample}}-{\mathrm{absorbance}}_{\mathrm{blank}}}{{\mathrm{absorbance}}_{\mathrm{control}}-{\mathrm{absorbance}}_{\mathrm{blank}}}\times 100$$

#### 3.2.3. Serum Stability

The serum stability assay was conducted to evaluate the effect of serum on the antimicrobial activity of NP-6 using a method that was reported in a previous study [41]. Peptide (1.0 mg/mL) was added to 50% fetal bovine serum and incubated at 37 °C for 0, 3, and 6 h, respectively. The cells that were incubated with PBS, NP-6, and serum were conducted as a blank and a control. The antimicrobial activity of NP-6 was measured using the plate count method. These assays were repeated three times independently. The inhibition rate of samples was calculated according to the following formula:

(3) $$\mathrm{Inhibition}\;\mathrm{rate}\;\left(\%\right)=\frac{N_{blank}-N_{sample}}{N_{blank}}\times 100$$

where *N_blank_* is the colony forming units (CFU) of cells that were incubated with PBS and *N_sample_* is the CFU of cells that were incubated with NP-6, NP-6+ serum, and serum, respectively. The inhibition rate of NP-6+ serum is expressed as the relative value to NP-6.

### 3.3. Observation of Transmission Electron Microscopy (TEM)

The TEM test was conducted according to Shi et al. [42] with some modifications. Briefly, log-phased cells of *S. aureus* were treated with NP-6 (1.0 mg/mL) for 2 h at 37 °C, followed by being centrifuged and washed three times with phosphate-buffered saline (PBS, 0.01 M, pH 7.4). The PBS-treated cells were set as the control. After incubation, the cells were centrifuged at 5000× *g*, 4 °C for 10 min with the supernatant discarded and then washed with PBS three times. The collected cells were fixed with 500 µL of 2.5% glutaraldehyde overnight, then post-fixed with 2% osmium tetroxide for 2 h. Both were conducted at 4 °C. The fixed cells were then washed twice with PBS, dehydrated with 30%, 50%, 70%, 80%, 90%, and 100% ethanol for 15 min each time. After dehydration, the samples were embedded in resin overnight and sliced. The cells that were treated with PBS were set as control. The analysis was conducted by a transmission electron microscopy system (Tecnai G2 F20 S-TWIN, FEI, Hillsboro, NH, USA).

### 3.4. Confocal Laser Scanning Microscopy (CLSM) Test

The assay was conducted by using PI staining combined with laser confocal microscope technology. Log-phased cells of *S. aureus* were collected and treated with NP-6 (1.0 mg/mL), and then the mixture was incubated at 37 °C for 2 h with shaking (160 rpm/min). After the incubation, the cells were washed twice with PBS. A working solution containing 1.5 µL SYTO 9 (5 mM in DMSO), 1.5 µL PI (50 µg/mL in dd H_2_O), and 1 mL PBS was prepared to stain *S. aureus*. Following incubation with the working solution at 4 °C for 30 min in the dark, the cells were observed under an SP2 confocal microscope (Leica, German) after washing twice with PBS. The cells that were treated with PBS were set as the control. The samples were illuminated with a laser at 485 nm for excitation and the emission was measured at 530 nm (SYTO9 emission wavelength) and 630 nm (PI emission wavelength). 

### 3.5. Flow Cytometric Analysis

The test was performed according to Joshi et al. [43] with some modifications. Log-phased cells of *S. aureus* were centrifuged (15,000× *g*, 4 °C, 5 min), washed, and resuspended in PBS to a final density of 1 × 10^6^ CFU/mL. NP-6 was added to the suspension to a final concentration of 1 mg/mL, followed by incubation at 37 °C for 1 h. After that, the *S. aureus* cells were collected, washed, and finally incubated with PI solution (50 µg/mL) at 4 °C for 30 min in the dark. The cells that were treated with PBS were set as the control. Unbound PI was removed by centrifugation (15,000× *g*, 4 °C, 5 min). The number of bacterial cells that were stained by PI was recorded by a FACSCalibur flow cytometer (Becton Dickinson, Franklin Lakes, NJ, USA).

### 3.6. Competitive Binding of NP-6 and EB with Bacterial DNA

Genomic DNA of *S. aureus* was prepared using bacterial genomic DNA extraction kit according to the instructions (Tiangen Biotech Co., Ltd., Beijing, China). The experiment was conducted as described by Li et al. [44] with some modifications. *S. aureus* genomic DNA was diluted to 50 µg/mL with TE buffer (10 mM, pH 8.0). A total of 50 µL DNA solution and 15 µL EB (100 µg/mL) were mixed in a sterilized 96-well plate and incubated at 37 °C for 10 min in the dark. NP-6 with different concentrations (0, 1.0, 2.0, and 4.0 mg/mL) was added to the mixture, followed by incubation at 37 °C for 30 min in the dark. The samples that were treated with PBS were set as the control. The analysis was conducted by a Lumina Fluorescence Spectrophotometer (Thermo Fisher Scientific, Waltham, MA, USA) with the excitation wavelength setting at 535 nm. The spectra were recorded at 550~750 nm.

### 3.7. DNA Gel Retardation

DNA gel retardation was conducted according to a previous method with little modification [45]. NP-6 was dissolved in DNA binding buffer which was composed of 5% glycerol, 10 mM Tris-HCl (pH 8.0), 1 mM EDTA, 1mM dithiothreitol (DTT), 20 mM KCl, and 50 µg/mL bovine serum albumin (BSA), and then diluted in different concentrations (0, 0.1, 0.2, 0.4, 0.8, 1.0, 2.0, and 4.0 mg/mL). A total of 5 µL DNA samples and 5 µL NP-6 were mixed in a 100 µL EP tube. The mixture was incubated at room temperature for 10 min and subjected to gel electrophoresis on a 1% agarose gel. The results were recorded by the UVP gel imaging system. The samples that were treated with PBS were set as the control.

### 3.8. RNA Gel Retardation

The assay was conducted according to a previous method [22]. The total RNA of *S. aureus* was prepared using RNAprep pure Cell/Bacteria Kit according to the instructions (Tiangen Biotech Co., Ltd., Beijing, China). NP-6 was dissolved in diethylpyrocarbonate (DEPC)–treated water to final concentration of 0, 0.1, 0.2, 0.4, 0.8, 1.0, 2.0, and 4.0 mg/mL. A total of 5 µL NP-6 solution and the same volume of bacterial RNA were mixed and incubated at room temperature for 10 min. The mixture with DEPC water instead of NP-6 was set as a control. The RNA gel retardation was conducted using a one-stop RNA electrophoresis kit according to the manufacturer’s recommendations (Tiandz Gene Tech Co., Ltd., Beijing, China) and recorded by the UVP gel imaging system.

### 3.9. Effect of NP-6 on the Activity of T-ATPase

The assay was performed using a previous method with minor modification [42]. Briefly, after incubating with NP-6 at 37 °C for 1 h, the bacteria was centrifuged (13,400× *g*, 5 min, 4 °C), washed, and resuspended in PBS (0.01 M, pH 7.4). The bacteria that were incubated with PBS was conducted as a control. Then, the samples were disrupted by ultra-sonication at 300 W for 5 min in an ice-bath. The total protein content was determined using a BCA protein assay kit (Nanjing Jiancheng Bioengineering Institute, Nanjing, China). The T-ATPase activity was determined using a T-ATPase test kit according to the recommendations of the manufacturer (Nanjing Jiancheng Bioengineering Institute, Nanjing, China). The samples were scanned by a microplate reader (Dinex Technologies Inc., Chantilly, VA, USA) at 636 nm. T-ATPase concentrations were expressed in U/mg protein.

### 3.10. Effect of NP-6 on the Activity of β-Galactosidase

The assay was conducted according to a previous method [22]. Log-phased cells of *S. aureus* were collected, centrifuged, and resuspended in M9 media. After incubation at 37 °C for 8 h, the cells were centrifuged at 13,400× *g* for 5 min, washed three times, and resuspended in PBS (0.01 M, pH 7.4). Then, NP-6 was added to concentrations of 0.5, 1.0, and 2.0 mg/mL. The mixture was incubated at 37 °C for 1 h, with PBS-treated cells as the control. After that, the culture was centrifuged (13,400× *g*, 4 °C, 20 min) and disrupted by ultra-sonication at 300 W for 5 min in an ice-bath. The supernatant was collected on ice after centrifugation (15,000× *g*, 4 °C, 20 min). A total of 20 µL of the bacterial suspension was mixed with 80 µL of ONPG (5 mM) in a standard 96-well plate. The activity of β-galactosidase was measured using β-galactosidase activity assay kit according to the recommendations of the manufacturer (Sangon Biotech Co., Ltd., Shanghai, China). The samples were scanned by a microplate reader (Dinex Technologies Inc., Chantilly, VA, USA) at 420 nm. The total protein content was determined using a BCA protein assay kit (Nanjing Jiancheng Bioengineering Institute, Nanjing, China). The β-galactosidase concentrations were expressed in U/mg protein.

### 3.11. Statistical Analysis

All the tests were repeated at least three times. Data analysis was performed using the software of SPSS (version 19.0, IBM SPSS, Armonk, NY, USA). The results are expressed as the means ± standard deviation. The significance of the differences between the treatments and the respective controls was determined based on Student’s *t*-test, and a value of *p* < 0.05 was considered statistically significant.

## 4. Conclusions

In this study, we confirmed that NP-6 inhibits the growth of *S. aureus* by multiple modes of action. It had little hemolysis activity, good serum stability, and negligible cytotoxicity towards RAW264.7 cells when the concentration was lower than 512 µg/mL. Further research showed that NP-6 could destroy the integrity of the cell wall and cell membrane of *S. aureus*. In addition, NP-6 can also bind with bacterial DNA and RNA, strongly inhibiting the intracellular β-galactosidase activity of *S. aureus*. Multiple targets for NP-6 in the cell membrane and intracellular macromolecules illustrate that it could be a novel option for the prevention and control of *S. aureus* infections.

## Data Availability

Not applicable.

## Supplementary Materials

The following supporting information can be downloaded at: https://www.mdpi.com/article/10.3390/ijms23147812/s1.

## References

[1] Wang G., Li X., Wang Z. (2016). APD3: The antimicrobial peptide database as a tool for research and education. Nucleic Acids Res..

[2] Hultmark D., Steiner H., Rasmuson T., Boman H. (1980). Insect Immunity. Purification and Properties of Three Inducible Bactericidal Proteins from Hemolymph of Immunized Pupae of Hyalophora cecropia. Eur. J. Biochem..

[3] Lee T.H., Hall K.N., Aguilar M.I. (2016). Antimicrobial Peptide Structure and Mechanism of Action: A Focus on the Role of Membrane Structure. Curr. Top. Med. Chem..

[4] Li S., Wang Y., Xue Z., Jia Y., Li R., He C., Chen H. (2021). The structure-mechanism relationship and mode of actions of antimicrobial peptides: A review. Trends Food Sci. Technol..

[5] Cardoso M.H., Meneguetti B.T., Costa B.O., Buccini D.F., Oshiro K.G.N., Preza S.L.E., Carvalho C.M.E., Migliolo L., Franco O.L. (2019). Non-Lytic Antibacterial Peptides That Translocate Through Bacterial Membranes to Act on Intracellular Targets. Int. J. Mol. Sci..

[6] Madrazo A.L., Campos M.R.S. (2020). Review of antimicrobial peptides as promoters of food safety: Limitations and possibilities within the food industry. J. Food Saf..

[7] Lee T.H., Hofferek V., Separovic F., Reid G.E., Aguilar M.I. (2019). The role of bacterial lipid diversity and membrane properties in modulating antimicrobial peptide activity and drug resistance. Curr. Opin. Chem. Biol..

[8] da Silva A., Teschke O. (2003). Effects of the antimicrobial peptide PGLa on live Escherichia coli. Biochim. Biophys. Acta (BBA)-Mol. Cell Res..

[9] Zamora-Carreras H., Strandberg E., Mühlhäuser P., Bürck J., Wadhwani P., Jiménez M.Á., Bruix M., Ulrich A.S. (2016). Alanine scan and 2 H NMR analysis of the membrane-active peptide BP100 point to a distinct carpet mechanism of action. Biochim. Biophys. Acta (BBA)-Biomembr..

[10] Matsuzaki K., Mechanisms M.P., Matsuzaki K. (2019). Antimicrobial Peptides: Basics for Clinical Application.

[11] Matsuzaki K., Yoneyama S., Murase O., Miyajima K. (1996). Transbilayer Transport of Ions and Lipids Coupled with Mastoparan X Translocation. Biochemistry.

[12] Kobayashi S., Chikushi A., Tougu S., Imura Y., Nishida M., Yano Y., Matsuzaki K. (2004). Membrane Translocation Mechanism of the Antimicrobial Peptide Buforin 2. Biochemistry.

[13] Huang Y., Huang J., Chen Y. (2010). Alpha-helical cationic antimicrobial peptides: Relationships of structure and function. Protein Cell.

[14] Laver D.R. (1994). The barrel-stave model as applied to alamethicin and its analogs reevaluated. Biophys. J..

[15] Ramamoorthy A., Lee D., Narasimhaswamy T., Nanga R.P.R. (2010). Cholesterol reduces pardaxin’s dynamics—A barrel-stave mechanism of membrane disruption investigated by solid-state NMR. Biochim. Biophys. Acta (BBA)-Biomembr..

[16] Nicolas P. (2009). Multifunctional host defense peptides: Intracellular-targeting antimicrobial peptides. FEBS J..

[17] Le C., Fang C., Sekaran S.D. (2017). Intracellular Targeting Mechanisms by Antimicrobial Peptides. Antimicrob. Agents Chemother..

[18] Graf M., Mardirossian M., Nguyen F., Seefeldt A.C., Guichard G., Scocchi M., Innis C.A., Wilson D.N. (2017). Proline-rich antimicrobial peptides targeting protein synthesis. Nat. Prod. Rep..

[19] Roy R.N., Lomakin I.B., Gagnon M.G., Steitz T.A. (2015). The mechanism of inhibition of protein synthesis by the proline-rich peptide oncocin. Nat. Struct. Mol. Biol..

[20] Li L., Sun J., Xia S., Tian X., Cheserek M.J., Le G. (2016). Mechanism of antifungal activity of antimicrobial peptide APP, a cell-penetrating peptide derivative, against Candida albicans: Intracellular DNA binding and cell cycle arrest. Appl. Microbiol. Biotechnol..

[21] Miao J., Zhou J., Liu G., Chen F., Chen Y., Gao X., Dixon W., Song M., Xiao H., Cao Y. (2016). Membrane disruption and DNA binding of Staphylococcus aureus cell induced by a novel antimicrobial peptide produced by Lactobacillus paracasei subsp. tolerans FX-6. Food Control..

[22] Hou X., Li S., Luo Q., Shen G., Wu H., Li M., Liu X., Chen A., Ye M., Zhang Z. (2019). Discovery and identification of antimicrobial peptides in Sichuan pepper (*Zanthoxylum bungeanum* Maxim) seeds by peptidomics and bioinformatics. Appl. Microbiol. Biotechnol..

[23] Aronica P.G.A., Reid L.M., Desai N., Li J., Fox S.J., Yadahalli S., Essex J.W., Verma C.S. (2021). Computational methods and tools in antimicrobial peptide research. J. Chem. Inf. Model..

[24] Pushpanathan M., Pooja S., Gunasekaran P., Rajendhran J. (2016). Critical evaluation and compilation of physicochemical determinants and membrane interactions of MMGP1 antifungal peptide. Mol. Pharm..

[25] Goudarzi F., Asadi A., Afsharpour M., Robab H.J. (2018). In vitro characterization and evaluation of the cytotoxicity effects of nisin and nisin-loaded PLA-PEG-PLA nanoparticles on gastrointestinal (AGS and KYSE-30), hepatic (HepG2) and blood (K562) cancer cell lines. AAPS PharmSciTech.

[26] Zhao J., Zhao C., Liang G., Zhang M., Zheng J. (2013). Engineering antimicrobial peptides with improved antimicrobial and hemolytic activities. J. Chem. Inf. Model..

[27] Sahariah P., Sørensen K.K., Hjálmarsdóttir M.Á., Sigurjonsson O.E., Jensen K.J., Masson M., Thygesen M.B. (2015). Antimicrobial peptide shows enhanced activity and reduced toxicity upon grafting to chitosan polymers. Chem. Commun..

[28] Hou X., Feng C., Li S., Luo Q., Shen G., Wu H., Li M., Liu X., Chen A., Ye M. (2019). Mechanismof antimicrobial peptide NP-6 from Sichuan pepper seeds against E. coli and effects of different environmental factors on its activity. Appl. Microbiol. Biotechnol..

[29] Xu L., Chou S., Wang J., Shao C., Li W., Zhu X., Shan A. (2015). Antimicrobial activity and membrane-active mechanism of tryptophan zipper-like β-hairpin antimicrobial peptides. Amino Acids.

[30] Wang C., Zhou Y., Li S., Li H., Tian L., Wang H., Shang D. (2013). Anticancer mechanisms of temporin-1CEa, an amphipathic α-helical antimicrobial peptide, in Bcap-37 human breast cancer cells. Life Sci..

[31] Song W., Kong X., Hua Y., Chen Y., Zhang C., Chen Y. (2020). Identification of antibacterial peptides generated from enzymatic hydrolysis of cottonseed proteins. LWT.

[32] Anunthawan T., de la Fuente-Núñez C., Hancock R.E.W., Klaynongsruang S. (2015). Cationic amphipathic peptides KT2 and RT2 are taken up into bacterial cells and kill planktonic and biofilm bacteria. Biochim. Biophys. Acta (BBA)-Biomembr..

[33] Liu D., Liu J., Li J., Xia L., Yang J., Sun S., Ma J., Zhang F. (2017). A potential food biopreservative, CecXJ-37N, non-covalently intercalates into the nucleotides of bacterial genomic DNA beyond membrane attack. Food Chem..

[34] Séverine D., Chiara G., Frédérique L., Heinz S., Vassilios I., Christine D. (2021). Expasy, the Swiss Bioinformatics Resource Portal, as designed by its users. Nucleic Acids Res..

[35] Kuriata A., Gierut A.M., Oleniecki T., Ciemny M.P., Kolinski A., Kurcinski M., Kmiecik S. (2018). CABS-flex 2.0: A web server for fast simulations of flexibility of protein structures. Nucleic Acids Res..

[36] Rousseau F., Schymkowitz J., Serrano L. (2006). Protein aggregation and amyloidosis: Confusion of the kinds. Curr. Opin. Struct. Biol..

[37] Lomize M.A., Pogozheva I.D., Joo H., Mosberg H.I., Lomize A.L. (2012). OPM database and PPM web server: Resources for positioning of proteins in membranes. Nucleic Acids Res..

[38] Tang W., Zhang H., Wang L., Qian H. (2014). New cationic antimicrobial peptide screened from boiled-dried anchovies by immobilized bacterial membrane liposome chromatography. J. Agric. Food Chem..

[39] Kim M.K., Kang N., Ko S.J., Park J., Park E., Shin D.W., Kim S.H., Lee S.A., Lee J.I., Lee S.H. (2018). Antibacterial and antibiofilm activity and mode of action of magainin 2 against drug-resistant acinetobacter baumannii. Int. J. Mol. Sci..

[40] Deng W., Liu K., Cao S., Sun J., Zhong B., Jiong C. (2020). Chemical composition, antimicrobial, antioxidant, and antiproliferative properties of grapefruit essential oil prepared by molecular distillation. Molecules.

[41] Wang C., Shao C., Fang Y., Wang J., Dong N., Shan A. (2021). Binding loop of sunflower trypsin inhibitor 1 serves as a design motif for proteolysis-resistant antimicrobial peptides. Acta Biomater..

[42] Shi W., Li C., Li M., Zong X., Han D., Chen Y. (2016). Antimicrobial peptide melittin against *Xanthomonas oryzae* pv. *oryzae*, the bacterial leaf blight pathogen in rice. Appl. Microbiol. Biotechnol..

[43] Joshi S., Bisht G.S., Rawat D.S., Kumar A., Kumar R., Maiti S., Pasha S. (2010). Interaction studies of novel cell selective antimicrobial peptides with model membranes and E. coli ATCC 11775. Biochim. Biophys. Acta (BBA)-Biomembr..

[44] Li L., Shi Y., Cheserek M.J., Su G., Le G. (2013). Antibacterial activity and dual mechanisms of peptide analog derived from cell-penetrating peptide against Salmonella typhimurium and Streptococcus pyogenes. Appl. Microbiol. Biotechnol..

[45] Nam J., Yun H., Rajasekaran G., Kumar S.D., Kim J.I., Min H.J., Shin S.Y., Lee C.W. (2018). Structural and Functional Assessment of mBjAMP1, an Antimicrobial Peptide from Branchiostoma japonicum, Revealed a Novel α-Hairpinin-like Scaffold with Membrane Permeable and DNA Binding Activity. J. Med. Chem..

