# Supplementary material for: Antibacterial Peptide NP-6 Affects Staphylococcus aureus by Multiple Modes of Action
Source: Int J Mol Sci. 2022 Jul 15;23(14):7812. doi: 10.3390/ijms23147812 (PMC9319634; doi:10.3390/ijms23147812)
Supplement: Supplementary file 1 [file ijms-23-07812-s001.zip › ijms-1770926-supplementary.pdf]

Antibacterial peptide NP-6 affects *Staphylococcus aureus* by multiple modes of action

Table S1. Prediction of physicochemical properties of NP-6.

**Properties**	**NP-6**
Theoretical isoelectric point	11.10
Net charge	+4
Half-life (Mammal)	44 h
Half-life (Yeast)	>20 h
Half-life (E. coli)	>10 h
GRAVY	-0.174
amphipathy	0.84

Note: GRAVY; Grand average of hydropathicity.

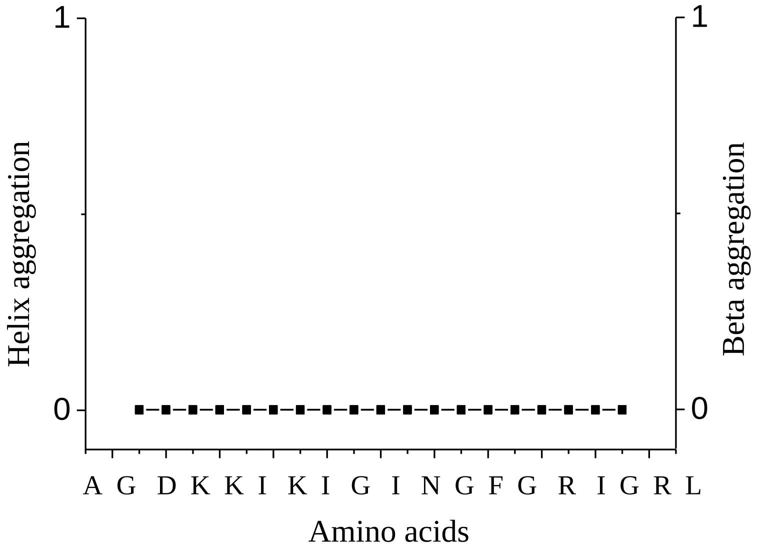

Figure S1. Prediction of aggregation potentiality of NP-6 in solution by Aggrescan 3D server

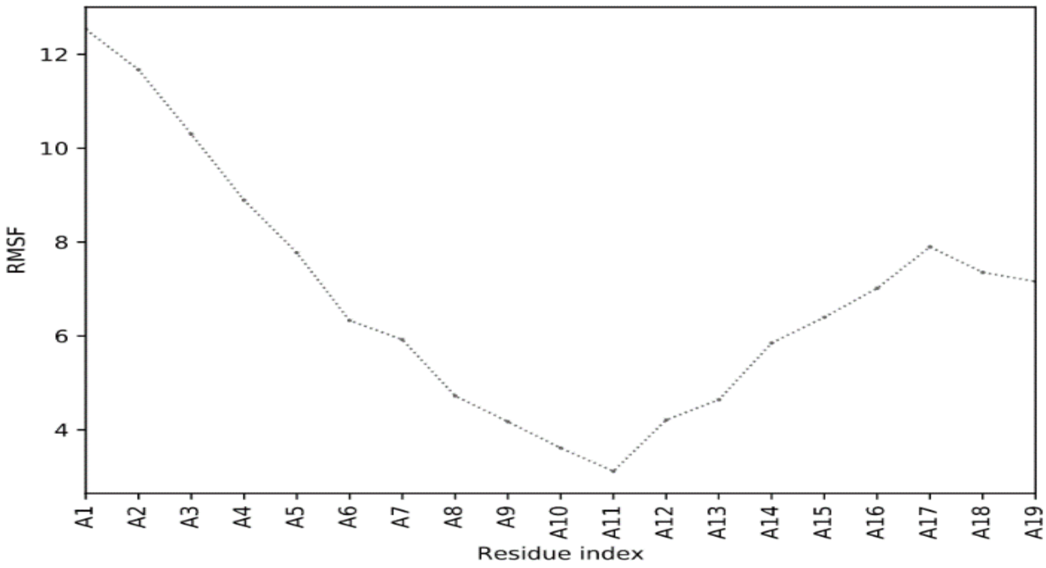

Figure S2. Prediction of aggregation potentiality of NP-6 in the presence of bacterial membrane by Tango.

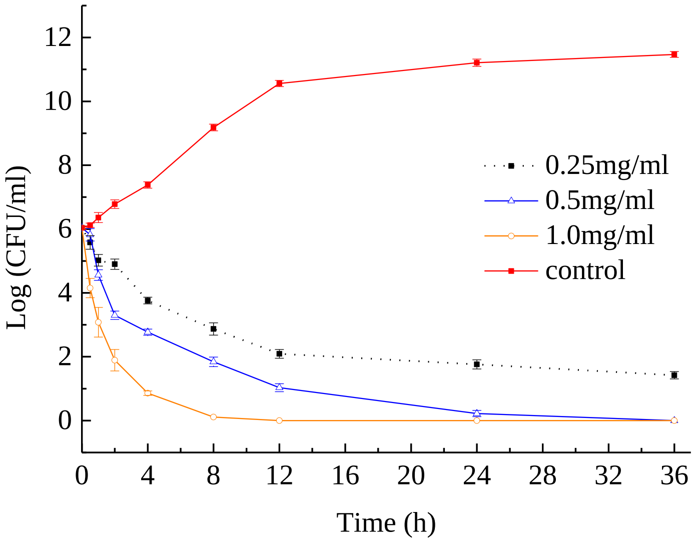

Figure S3. Time-killing curve of NP-6 against *S. aureus*

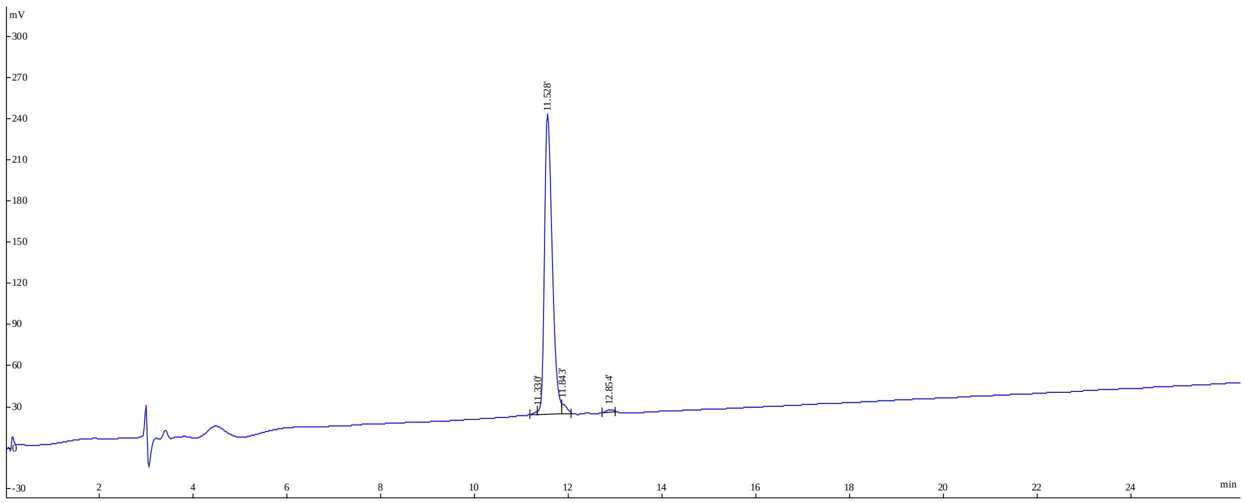

(a)

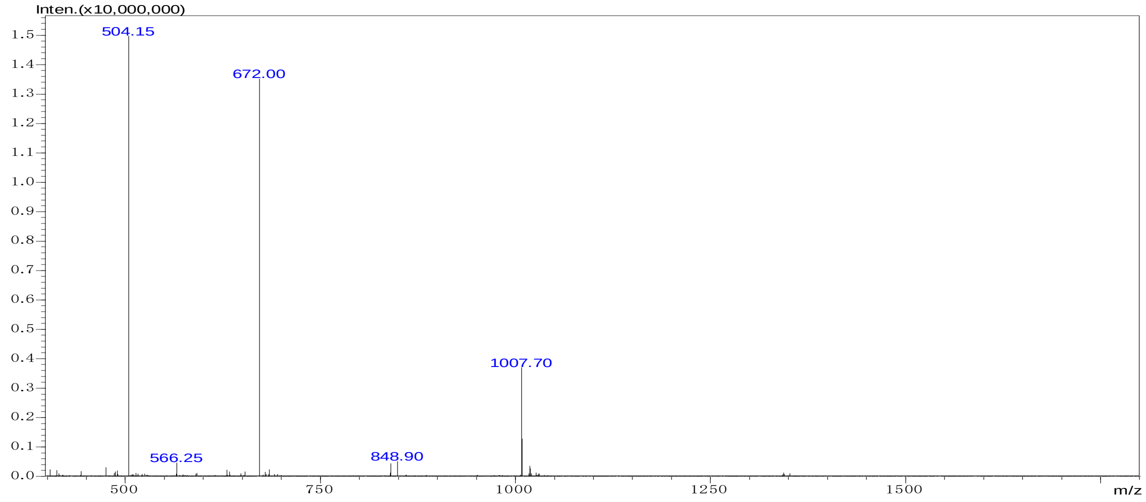

(b)

Figure S4. High performance liquid chromatography (a) and mass spectrometry (b) of NP-6
